# Unveiling the molecular landscape of cognitive aging: insights from polygenic risk scores, DNA methylation, and gene expression

**DOI:** 10.1186/s40246-024-00640-6

**Published:** 2024-07-02

**Authors:** Sonya Neto, Andreia Reis, Miguel Pinheiro, Margarida Ferreira, Vasco Neves, Teresa Costa Castanho, Nadine Santos, Ana João Rodrigues, Nuno Sousa, Manuel A. S. Santos, Gabriela R. Moura

**Affiliations:** 1 Institute for Biomedicine (iBiMED) and Department of Medical Sciences, University of Aveiro, 3810-193 Aveiro, Portugal; 2 ICVS - School of Medicine, Campus Gualtar, University of Minho, 4710-057 Braga, Portugal; 3 Clinical Academic Center – Braga (2CA-B), Braga, Portugal; 4 P5 Medical Center, Braga, Portugal; 5 Multidisciplinary Institute of Aging, MIA-Portugal, Faculty of Medicine, University of Coimbra, Rua Largo 2, 3º, 3000-370 Coimbra, Portugal

**Keywords:** Cognitive aging, Cerebral small vessel disease (CSVD), Polygenic risk scores (PRS), Multi-omics data integration, Age-related methylation alterations, Age-related transcriptomic alterations

## Abstract

**Background:**

Aging represents a significant risk factor for the occurrence of cerebral small vessel disease, associated with white matter (WM) lesions, and to age-related cognitive alterations, though the precise mechanisms remain largely unknown. This study aimed to investigate the impact of polygenic risk scores (PRS) for WM integrity, together with age-related DNA methylation, and gene expression alterations, on cognitive aging in a cross-sectional healthy aging cohort. The PRSs were calculated using genome-wide association study (GWAS) summary statistics for magnetic resonance imaging (MRI) markers of WM integrity, including WM hyperintensities, fractional anisotropy (FA), and mean diffusivity (MD). These scores were utilized to predict age-related cognitive changes and evaluate their correlation with structural brain changes, which distinguish individuals with higher and lower cognitive scores. To reduce the dimensionality of the data and identify age-related DNA methylation and transcriptomic alterations, Sparse Partial Least Squares-Discriminant Analysis (sPLS-DA) was used. Subsequently, a canonical correlation algorithm was used to integrate the three types of omics data (PRS, DNA methylation, and gene expression data) and identify an individual “omics” signature that distinguishes subjects with varying cognitive profiles.

**Results:**

We found a positive association between MD-PRS and long-term memory, as well as a correlation between MD-PRS and structural brain changes, effectively discriminating between individuals with lower and higher memory scores. Furthermore, we observed an enrichment of polygenic signals in genes related to both vascular and non-vascular factors. Age-related alterations in DNA methylation and gene expression indicated dysregulation of critical molecular features and signaling pathways involved in aging and lifespan regulation. The integration of multi-omics data underscored the involvement of synaptic dysfunction, axonal degeneration, microtubule organization, and glycosylation in the process of cognitive aging.

**Conclusions:**

These findings provide valuable insights into the biological mechanisms underlying the association between WM coherence and cognitive aging. Additionally, they highlight how age-associated DNA methylation and gene expression changes contribute to cognitive aging.

**Supplementary Information:**

The online version contains supplementary material available at 10.1186/s40246-024-00640-6.

## Background

Cognitive aging is depicted by a decline in several cognitive domains, mainly in executive function, memory, and information processing speed [1] and each of these cognitive abilities has been associated with morphometric changes in specific neuroanatomic regions [2, 3]. Moreover, cognitive aging seems to result from inefficient communication within neurocognitive networks such as the frontal-striatal network [4]. Thus, white matter (WM) integrity seems critical for proper cognitive function [5]. Magnetic resonance imaging (MRI) markers, including WM hyperintensities (WMH), Fractional Anisotropy (FA), and Mean Diffusivity (MD), can detect WM changes and provide quantitative measures to assess its integrity. With advancing age, WM undergoes natural age-related changes. These changes include increased WMH load, reduced FA, and increased MD, suggesting a decline in white matter integrity [6]. In fact, WM lesions represent a risk factor for several health conditions, including stroke, vascular dementia, and age-related cognitive impairment [7]. The pathogenesis is not fully understood but it is presumed to have a vascular origin, resulting from ischemic injury or related to endothelial dysfunction and age-associated blood–brain barrier (BBB) disruptions [8]. Like many other age-associated traits and frequent comorbid complex diseases, it is possible that the phenotypic associations between WM integrity and age-related cognitive alteration are a result of widespread pleiotropic effects and thus, partly mediated by a shared genetic etiology [9, 10].

A common approach to test shared genetic effects is to calculate polygenic risk scores (PRS) which are estimated by summing the risk alleles of a phenotype of interest weighted by the effect size derived from the most robust genome-wide association study (GWAS) on the phenotype [11]. The method was first applied to evaluate shared genetic variants in schizophrenia and bipolar disorder [12] and, since then, PRS have been used to test whether the genetic architecture of many comorbid diseases and endophenotypes overlaps. PRS can be applied to two different traits making it possible to test the genetic propensity of an individual to a wide range of diseases. For example, Mclntosh et al. [13] showed that PRS for schizophrenia predicts the development of cognitive deficits in older ages and others used PRS to demonstrate that the phenotypic correlation between coronary artery disease and cognitive ability is mediated by shared genetic effects [14].

GWAS have successively found associations of genetic variants to several diseases. Nevertheless, nearly 90% of disease-associated variants are in non-protein-coding regions suggesting that many of these variants might be affecting disease risk by altering gene regulation [15]. DNA methylation is a common epigenetic mechanism regulating gene expression. Also, age-dependent methylation changes have been used as an effective biomarker to predict biological age and a promising biomarker to trace age acceleration in age-related diseases [16]. Therefore, age-related methylation changes and resulting gene expression alterations might be associated with the pathogenesis of major age-associated diseases. However, the study of transcriptomic and methylation data in the central nervous system is limited by sample availability, largely because brain tissue requires post-mortem collection, and the acquisition of cerebrospinal fluid samples require invasive procedures. Conversely, peripheral blood mononuclear cells (PBMCs) are easily acquired, and studies show that molecular alterations in PBMCs may serve as surrogates or biomarkers for CNS disorders [17].

Categorized into twelve interconnected hallmarks [18] the aging processes largely intersect with many molecular mechanisms underlying many age-related diseases which in a way validates epidemiological comorbidity and overlapping symptomology and, highlights shared pathophysiology [9]. Determining the underlining molecular pathways affected by age and implicated in disease susceptibility is a critical milestone to enable better medical healthcare to treat a growing aged population. Moreover, by better understanding the effects of age-related changes in cognition we can assess the effects of pathological disease states. Therefore, the aim of this work was two-fold. First, we hypothesized that the phenotypic associations between WM integrity and age-related cognitive alterations have shared genetic etiology. Second, to grasp a better understanding of the molecular link between aging and age-related cognitive performance by assessing how age-associated methylation and transcriptomic changes related to cognitive aging.

## Results

### Age-related cognitive changes and associated structural brain alterations

**Fig. 1:**
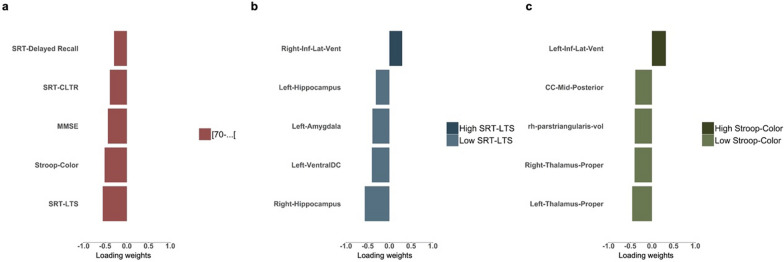
Age-related cognitive alteration and associated structural brain alteration. Loading plots showing cognitive tests that best discriminate age group ([50–60], [60–70], [70-…]) (**A**) and top 5 structural MRI features best-discriminating individuals with higher and lower cognitive scores in SRT-LTS (**B**) and Stroop-Colors (**C**). The plots display the weights of each selected variable on the first component of the sPLS-DA model, with color indicating the class with the minimum mean value

sPLS-DA analysis was first performed on neurocognitive tests using data from the Minho aging cohort (descriptive statistics are provided in Table 1) to determine individual cognitive tests best discriminating [50–60[, [60–70] and [70-…] age groups. sPLS-DA is particularly suited for this analysis because it handles multicollinearity effectively, which is common among cognitive tests. Moreover, it reduces the dimensionality of the data and focus on the cognitive tests that contribute most significantly to the discrimination between age groups. By identifying a sparse set of features that contribute most significantly to class discrimination, sPLS-DA ensures that the selected cognitive tests are the most relevant for distinguishing between age groups. Parameter tuning for the number of components onto which the data is projected and the optimal number of features to select on each component were determined by tuning these parameters using a cross-validation procedure (as described in the materials and methods section). Among the cognitive tests, STR-LTS, Stroop Color Test, MMSE, SRT-CLTR, and SRT-Delayed-Recall were selected as the most effective at discriminating between different age groups in terms of cognitive performance (Fig. 1a and Supplementary Table 1). Loading weights, depicted in Fig. 1 and Supplementary Table 1, reflect the contribution of each test to the separation of the age groups, indicating the importance of each test in distinguishing between the age groups. The oldest age group (> 70) exhibited lower mean values in all five cognitive tests, indicating worsening performance in cognitive tasks with age (Fig. 1a and Supplementary Table 1). The SRT-LTS and Stroop-Color Test presented the higher loadings weights (− 0.557 and − 0.511, respectively), meaning that they were the most relevant cognitive variables discriminating the different age groups. Next, sPLS-DA was applied to imaging data towards identifying structural brain alteration best-discriminating individuals with higher and lower cognitive scores in the SRT-LTS and Stroop-Color test. For the SRT-LTS, parameter tuning established an optimal number of 2 components with 10 and 5 imaging variables in each component, respectively (Supplementary Table 2a–b). On the other hand, 4 components and 10,10,5, and 25 imaging variables in each component, respectively, were necessary to discriminate individuals with higher and lower scores in the Stroop-Color test (Supplementary Table 3a–d). The 5 top contributing imaging variables, on the first component, discriminating participants with higher and lower scores in the STR-LTS and the Stoop-Color are presented in Fig. 1b and c, respectively.

**Table 1:** Descriptive statistics of the Minho aging cohort

	[50–60] (*N* = 128)	[60–70] (*N* = 148)	[70-… ] (*N* = 166)	Total (*N* = 442)	*p* value
Gender					7.208e−01 1
F	70 (54.7%)	76 (51.4%)	83 (50.0%)	229 (51.8%)	
M	58 (45.3%)	72 (48.6%)	83 (50.0%)	213 (48.2%)	
Schoolyrs					1.662e−10 ^2^
	5.406				
Mean (SD)	(2.855)	4.243 (2.732)	3.175 (2.706)	4.179 (2.897)	
	0.000–		0.000		
Range	16.000	0.000–17.000	17.000	0.000–17.000

### The contribution of PRS for white matter integrity to cognitive and brain aging

**Fig. 2:**
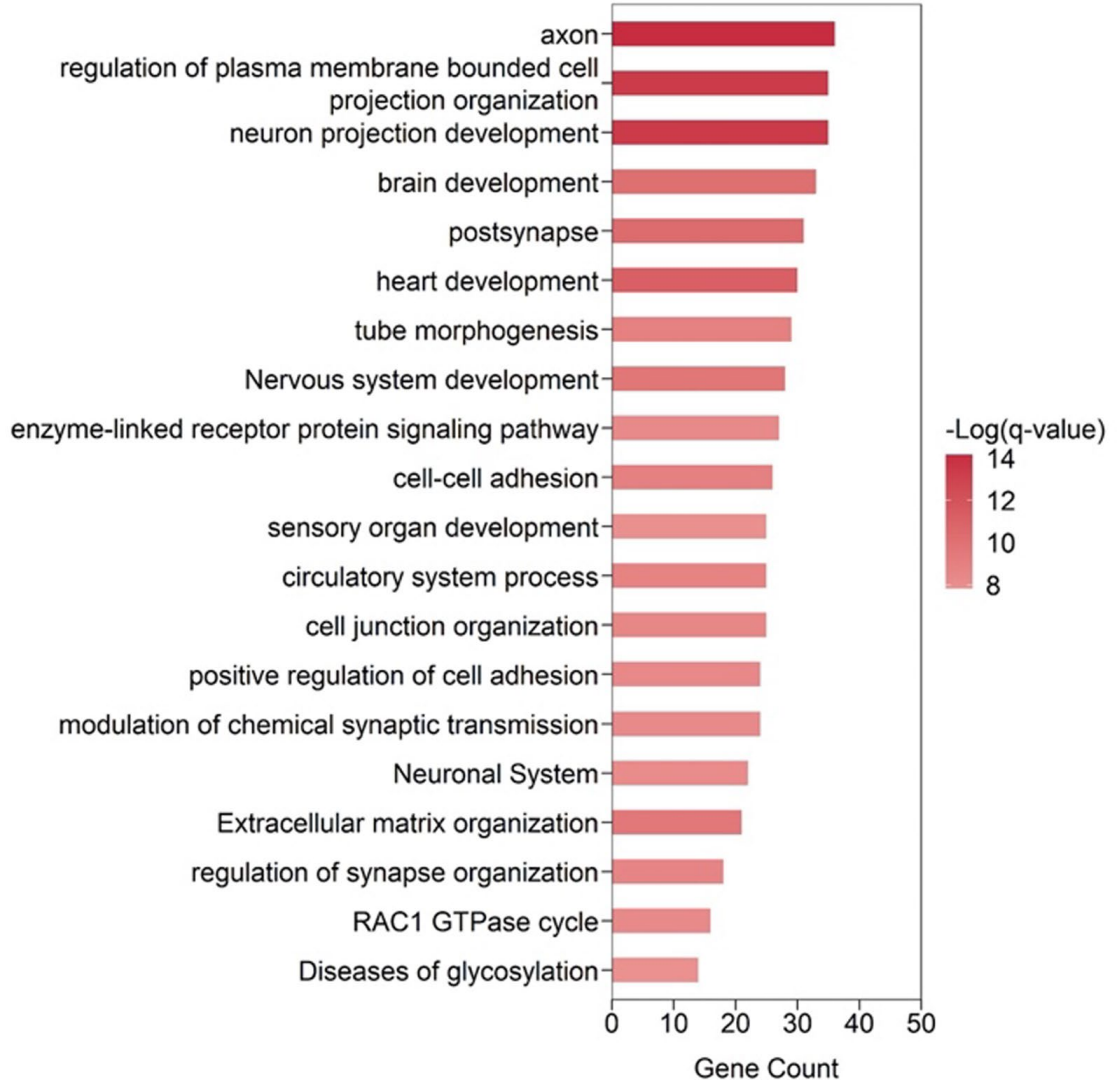
MD-PRS gene-set enrichment analysis. Top 20 clusters with their representative enriched terms (most significant term within each cluster). Log(q-value) is the multi-test adjusted p-value in log base 10

Genome-wide PRS analysis was performed to test associations between WMH, FA, MD, and the pre-selected age-related cognitive phenotypes (STR-LTS and Stroop Color Test). Nominal associations were found for WMH-PRS with Stroop colors (PRS.R^2^ = 1,1%, *p*-value = 0.01, at best Pt), MD PRS with STR/LTS (PRS.R^2^ = 2,3%, p-value = 0,001, at best Pt), and FA with STR/LTS (PRS.R2 = 1.2%, *p*-value = 0,01, at best Pt). Results for best-fitting p-value threshold are presented in Table 2. In the analysis of the association between the PRSs and cognitive outcomes only MD-PRS for STR-LTS passed FDR correction for multiple testing (FDR *p* = 0.04). Next, we tested whether besides being associated with SRT-LTS, MD-PRS scores were correlated to structural brain alterations, as shown in Table 3, discriminating individuals with lower and higher scores in SRT-LTS. We found that MD-PRS was positively correlated with WMH and negatively correlated with the left hemisphere amygdala, the right hemisphere fusiform white mater (rh-fusiform wm) and right hemisphere Temporal pole white matter, although only WMH and left amygdala results survived multi-comparison corrections (Table 3). Finally, to grasp a better understanding of the biological function coming from the polygenic signal we performed an enrichment analysis of the 1383 SNPs used in the construction of the polygenic model. A total of 190 gene sets were significantly enriched (Supplementary Table 4). These were grouped into 20 clusters (as described in material methods section), and the most significant term within each cluster was selected to represent the entire cluster (Fig. 2). This analysis revealed an enrichment of genes involved in brain development and structural plasticity (e.g. axon, regulation of plasma membrane bounded cell projection organization, neuron projection development, brain development, post-synapse, nervous system development, modulation of chemical synaptic transmission, regulation of synapse organization, RAC1 GTPase cycle), circulatory system development/process (e.g. heart development, tube morphogenesis, circulatory system process), cell adhesion/communication (e.g. cell–cell adhesion molecule binding, enzyme-linked receptor protein signaling pathway), immune and inflammatory response (positive regulation of cell adhesion) and extracellular matrix organization.

**Table 2:** Results of genome-wide PRS-analysis assessing genetic association between white matter hyperintensities (WMH), mead diffusivity (MD), fractional anisotropy (FA), and the Minho aging neurocognitive phenotypes controlling for age, sex, school years and 10 principal components

Base-Phenotyf)e	Target·Phenot pe	Threshold	PRS.R2	Full.R2	Null.R2	Coefficient	Standard.Error	*p*	Num_SNP	Empirical·p	FDR
WMH	STL-LTS	0,00395	0,003	0,232	0,228	252,052	188,901	0,183	3002	0,847	1,0000
WMH	Stoop-Colors	0,0004	0,011	0,315	0,304	166,778	66,072	0,012	502	0,135	0,4050
MD	STL-LTS	0,0013	0,023	0,251	0,228	− 82,216	23,544	0,001	1383	0,007	0,0400
MD	Stoop-Colors	0,0006	0,002	0,305	0,304	− 15,776	16,778	0,348	795	0,969	0,9690
FA	STL-LTS	0,0002	0,012	0,24	0,228	27,769	11,222	0,014	354	0,147	0,2950
FA	Stoop-Colors	0 00005	0004	0 308	0304	9 216	6281	0143	149	0 731	1 0000

Results are shown for the best-fitting genome-wide *P*-value threshold. *SNP* Single-nucleotide polymorphism, *Num_SNP* Number of SNPs included in the model, *PRS.R2* Variance explained by the PRS, *Full.R2* Variance explained by the full model (including the covariates), *Null.R2* Variance explained by the covariates, *SE* Standard error;. the *p*-value indicates nominal association of the model fit, *FDR* False Discovery Rate, Empirical *p* indicate the level of association and were adjusted for overfitting due to testing multiple *p*-value thresholds (based on 10,000 phenotypic permutations)

**Table 3:** Pairwise Spearman correlation analysis between MD-PRS and structural MRI features (components 1–2) best-discriminating individuals with higher and lower SRT-LTS scores

Pairwise correlations with MD-PRS	Correlation coefficient	*p*-value	q-value(FDR)
Left-Amygdala	− 0.298	0.006	0.044
White Matter Hyperintensities	0.301	0.005	0.044
rh-fusiform (wm)	− 0.269	0.014	0.068
rh-temporal pole (wm)	− 0.254	0.020	0.074
Right-Accumbens-area	− 0.219	0.045	0.135
Left-Inf-Lat-Vent	0.208	0.058	0.145
Left-Hippocampus	− 0.169	0.125	0.261
lh-inferior temporal (wm)	− 0.163	0.139	0.261
Right-Hippocampus	− 0.132	0.231	0.386
Left-Ventral DC	− 0.112	0.309	0.464
lh-precentral-vol	− 0.081	0.464	0.632
Right-Inf-Lat-Vent	0.061	0.581	0.727
rh-lateral occipital (wm)	− 0.052	0.641	0.740
rh-caudal middle frontal-vol	− 0.044	0.692	0.742
rh-lingual-vol	− 0,014	0.897	0.897

Nominally significant results (*p*-value < 0.05). *FDR* False discovery rate, *wm* White matter, *lh* Left hemisphere, *rh* Right hemisphere, *Inf* Inferior, *Lat* Lateral, *Vent* Ventricle *DC* Diencephalon, *CC* Corpus callosum, *vol* Volume

### Age-related methylation and transcriptomic alterations

**Fig. 3:**
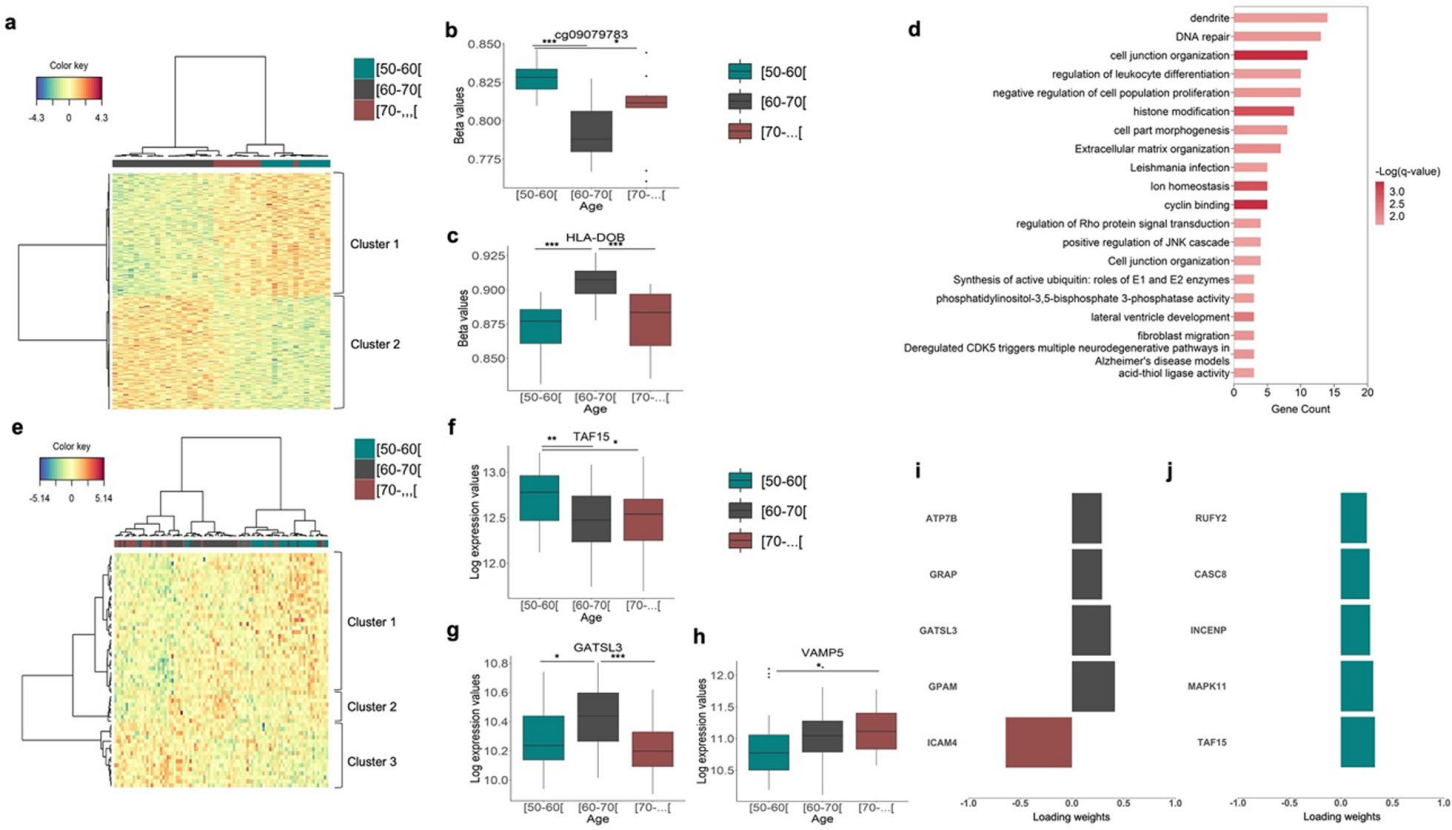
Age-related methylation and transcriptomic alterations. Heat map of selected CpG (**a**) and Genes (**e**) by sPLS-DA with samples in columns colored in turquoise, grey and raspberry representing [50–60], [60–70] and [70-…] age groups, respectively. The color key represents a spectrum of lowest expression (blue) to highest expression (red). Boxplots representing trends of GpG in cluster 1 (**b**) and cluster 2 (**c**). Gene-set enrichment analysis of methylation data (**d**) showing top 20 clusters with their representative enriched terms (most significant term within each cluster). Log(q-value) is the multi-test adjusted p-value in log base 10. Gene expression trends representing cluster 1, cluster 2, and cluster 3 (**e**) are presented in the boxplots **f**,** g** and **h**, respectively. The top 5 variables selected the best-discriminating age group ([50–60], [60–70], [70-…]) within transcriptomic data using the first (**i**) and second component (**j**). The plots display the loading weights of each selected variable of the sPLS-DA model, with color indicating the class with the maximum mean value

With the focus on identifying CpG sites and gene expression probes displaying age-related methylation and transcriptomic alterations, sPLS-DA was, separately, modeled using beta values of 7,346,680 CpG and 23,496 gene expression probes across 41 and 94 samples, respectively (Subsamples characteristics are present in Supplementary Tables 5 and 6).Parameter tuning indicated an optimal selection of 1 component and 350 CpG and 2 components and 58 gene expression probes, 8 in the first component and 50 in the second component, (refer to methods section and supplementary Table 6 and Table 8 a-b, respectively, for the full list of selected CpG and genes). Figure 3a displays the clustering of the methylation analysis, where two main clusters with similar profiles were identified. Custer 1 included CpG that were hypermethylated in the younger and older age groups while cluster 2, was composed of hypomethylated CpG in younger and older age groups. Of note, younger (50–60) and older age groups ([70-…]) tended to cluster together, displaying similar levels of methylation, while the middle age group [60–70] displayed a different pattern of methylation (Fig. 3a).

Methylation profiles seem to display non-linear relations with chronological age. In Figs. 3b and c, boxplots are presented to illustrate the trends of GpG methylation levels for specific methylation markers within the two distinct clusters identified in the analysis (Fig. 3a). These clusters are characterized by different patterns of methylation changes with respect to chronological age. Figure 3b represents the trend observed in cluster 1, displays a decreasing trend in methylation levels with age, reaching minimum levels in the [60–70] age group, followed by an increase in the [70-…] age group. Figure 3c represents the trend observed in cluster 2, with methylation levels increasing with age picking at the [60–70] age group, followed by a decrease in the [70-…] age group. These findings suggest non-linear relationships between methylation profiles of specific markers and chronological age”. Subsequently, we carried out pathway enrichment analysis of the selected variables. Annotated CpG were mapped to 164 gene-set, of which 99 were significantly enriched (q value < 0.05) (Supplementary Table 8), and they were grouped into 20 clusters (as described in material methods section), with the most significant term within each cluster being selected to represent the entire cluster (Fig. 3d). We identified an enrichment of several gene sets known to be associated with aging, including gene sets involved in intercellular communication (e.g. cell junction organization), genomic instability (e.g. DNA repair), proteostasis (e.g. synthesis of active ubiquitin: roles of E1 and E2 enzymes), cellular senescence (e.g. negative regulation of cell population proliferation) and immune response (e.g. regulation of leukocyte differentiation).

Heatmap plots of the transcriptomic data can be seen in Fig. 3e, where three clusters were identified. Cluster 1 included the genes that were mainly overexpressed in [50–60] and [60–70] age groups, Cluster 2 comprised genes overexpressed in [70-…[ age group, and Cluster 3 was composed of a subset of genes mainly overexpressed in [60–70[ age group. Boxplots in Figs. 3f–h further illustrate the trends of gene expression levels for specific genes within these clusters. Figure 3f displays the gene expression pattern observed in Cluster 1, Fig. 3g showcases the gene expression trend identified in Cluster 2, and Fig. 3h demonstrates the gene expression trend observed in Cluster 3. Thus, indicating both linear and non-linear relationship between gene expression and age.” As previously mentioned, parameter tuning determined an optimal selection of 2 components and 58 gene expression probes, 8 in the first component and 50 in the second component (please refer to information provided in the methods section and supplementary Table 7 a-b for the full list of selected genes). These components capture the most significant sources of variability within the data, notably discriminating between different age groups. The top 5 contributing genes from the first and second components are depicted in Fig. 3I–J, respectively.

### Multiomics data integration for age-related cognitive alteration

**Fig. 4:**
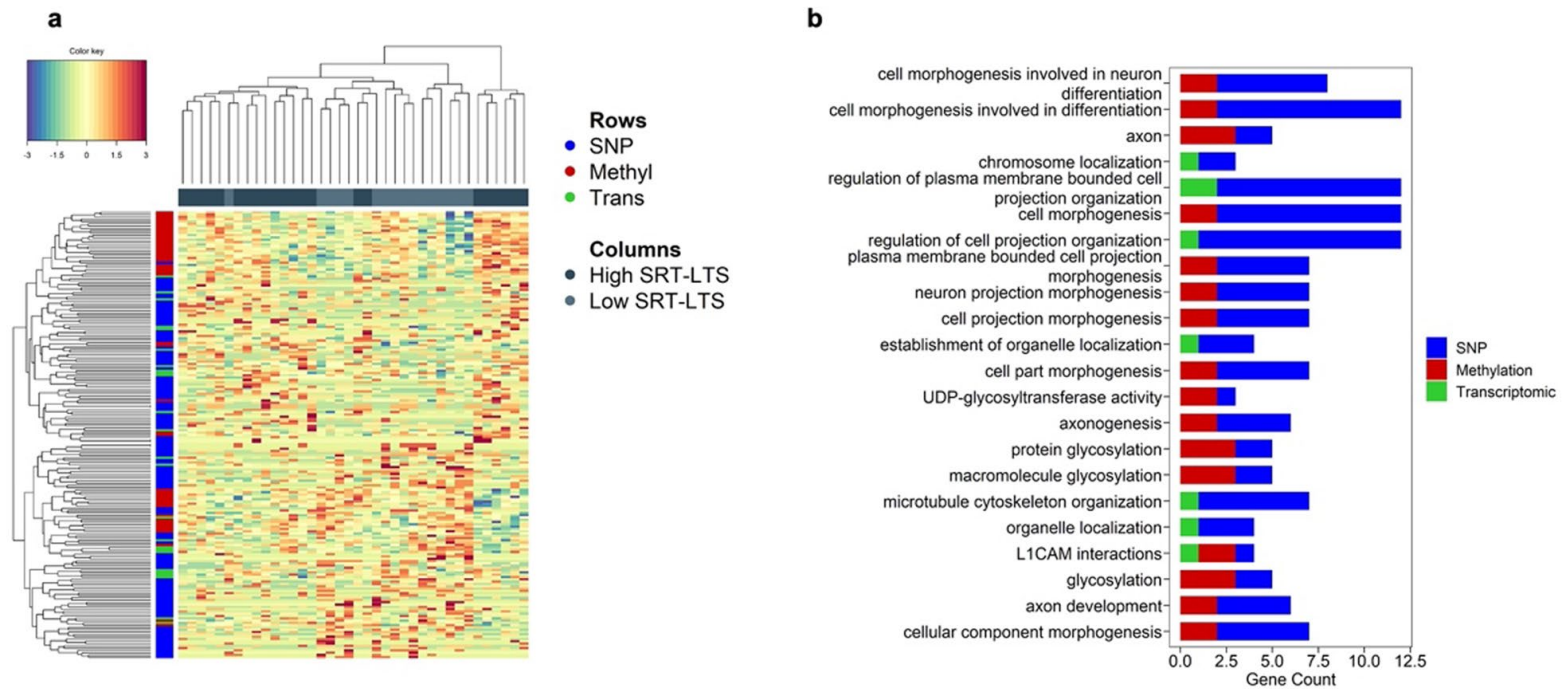
Multi-omic data integration for age-related cognitive alteration. Heat maps show the relationship between variables and each omics data block (**A**) with samples in columns colored in dark blue and light blue representing individuals with high and low scores in SRT-LTS, respectively. The different omics layers are represented as rows colored in blue, red, and green representing SNP, Methylation, and transcriptomic features, respectively. Gene-set enrichment analysis on the multi-omics signature (**B**). The length of the bar represents the number of genes in each ontology term, while the color represents the contribution of each omics with blue, red, and green representing SNP, CpGs and genes, respectively

Having identified major age-related methylation and transcriptomic alterations, our next objective was to explore how these changes, in conjunction with SNP (selected from the MD-PRS), could potentially impact cognitive aging. To accomplish that, we employed the DIABLO model, integrated into the MixOmics R package [19]. The DIABLO is specifically designed for the integrative analysis of multi-omics data aiming to identify a common latent structure that captures the shared information between multiple omics datasets in a supervised way. It does this by integrating the different datasets and finding a low-dimensional representation that maximizes the covariance between them. In this study, the DIABLO was modeled using the 350 CpG and 58 gene expression probes, previously selected as discriminating the different age groups, and 1383 SNPs, used in the construction of the MD-PRS model to identify a set of correlated variables that best discriminate individuals with higher and lower performance in the SRT-LTS memory test. After fine-tuning the model parameters (refer to methods section) a sub-set of 120 SNPs, 50 CpG sites, and 25 gene expression probes, projected into a single component, were selected. The clustering of the 38 samples, together with the selected variables, is depicted in Fig. 4a (refer to Supplementary Table 9 a-c for the complete list). Subsequently, the selected variables underwent pathway enrichment analysis using the web based Metascape porta [20]. Notably, this analysis revealed a significant enrichment of 22 terms (q value < 0.05), as shown in Fig. 4b (refer to Supplementary Table 10 for the complete list). Importantly, these enriched terms were associated with genes involved in pivotal processes such as synaptic dysfunction, axonal degeneration, microtubule cytoskeleton organization, and glycosylation.

## Discussion

### Age-related cognitive changes and associated structural brain alterations

While memory loss is one of the earliest cognitive alterations in aging, a decline in processing speed is pointed out as a leading indicator and critical mechanism behind age-related cognitive alterations [5, 21]. In line with this, we found that the most relevant age-related cognitive alterations were identified in cognitive variables of verbal learning and memory (STR-LTS) and processing speed (Stroop-Color Test). When analyzing structural brain alterations discriminating individuals with poorer and higher memory and processing speed scores, we found common and distinct brain structural alterations. Generally, participants with lower cognitive scores showed reduced hippocampus and amygdala volumes while showing enlargement of the inferior lateral ventricle and greater WM hyperintensities load. Interestingly, decreased volume of the hippocampus and amygdala together with an increased size of the inferior lateral ventricle has been associated with aging as well as Alzheimer’s Disease (AD) [22]. Moreover, when trying to dissociate structural brain alterations discriminating AD from normal aging, Coupé [22] and colleagues showed that AD brains start to diverge from normal brain aging before 40 years old and that this difference is enhanced with age. Additionally, our results are consistent with other studies that showed an association between memory performance and temporal lobe structures. Moreover, they support previous studies showing a positive relationship between the hippocampus size and memory in aging 2 and provide further evidence for the role of the hippocampus in other cognitive domains [23, 24] specifically in processing speed [25].

### The contribution of PRS for white matter integrity to cognitive and brain aging

There is strong evidence supporting a link between white matter integrity and age-related changes in cognitive abilities [6], and it is plausible that the observed phenotypic associations are influenced by shared genetic factors and common biological mechanisms. In this study, we found that individuals with a higher genetic risk for reduced white matter integrity exhibited lower age-sensitive memory scores, suggesting that these traits are influenced by common genetic factors, and corroborating other studies finding a phenotypic correlation between white matter integrity and age-related cognitive alterations [6].

Taken together, these findings indicate that being at high genetic risk of small vessel disease is associated with age-related memory decline and agree with other studies demonstrating a correlation between memory scores and diffusion tension imaging findings [26], mean diffusivity and WMH [27] as well as associations between temporal white matter microstructure and memory performance [28]. The mechanisms behind these associations might be multifaceted and likely mediated by genetic susceptibility to both vascular and non-vascular effects since our analysis point to enrichment in both circulatory system development/process and brain development and structural plasticity as well as cell adhesion/communication, immune and inflammatory responses and extracellular matrix organization**.** Moreover, common molecular mechanisms and pleiotropic genetic effects shared by both nerve and blood vessels might be major players in mediating these associations [29]. For example, several growth factors that promote blood vessel formation have been found to play important roles in regulating synaptic plasticity. For instance, vascular endothelial growth factor (VEGF) has been shown to enhance hippocampal long-term potentiation (LTP), a cellular mechanism underlying learning and memory processes [30].

### Age-related methylation and transcriptomic alterations

While age-related alteration typically exhibits a linear pattern, it is noteworthy that they can occur in a nonlinear pattern, meaning that changes do not adhere to a straightforward linear progression. For example, the effect of age on WM follows a non-linear pattern growing until 40–50 years before a steep reduction from 70 years of age onwards [31]. Similarly, it is estimated that the heritability of cognition increases through the lifespan, peaking in late adulthood and decreasing after 65 years of age [32]. Furthermore, in recent years several studies have highlighted the use of nonlinear models to access changes in gene expression 33, methylation [34, 35], and protein expression [36] during aging. Our results are consistent with these studies showing non-linear patterns of age-related methylation and transcriptomic alterations. Notably, our analysis established that the younger and the older group displayed similar methylation profiles with most abrupt changes occurring in the middle-aged group [60–70], a pattern also observed in a subset of genes in the transcriptomic analysis. Nonlinear patterns of age-related alteration can be influenced by a variety of factors, such as genetics, lifestyle, environmental factors, and gene-by-environment interactions as well as being possibly related to buffering mechanisms [37]. Gene-set enrichment analysis of selected CpGs identified many gene sets related to key hallmarks of aging [18], including genomic instability (e.g., DNA repair), altered intercellular communication (e.g., cell junction organization, extracellular matrix organization), inflammation (e.g., regulation of leukocyte differentiation), and epigenetic alterations (e.g., histone modification).

Moreover, among the top transcripts (from the first and second components) discriminating the 3 age groups, there were genes implicated in key signaling pathways that regulate aging and promote lifespan, many of which have been implicated in cellular senescence. For example, the ICAM4 is a gene that encodes a protein involved in cell–cell adhesion, which is critical for the immune system's proper function, allowing immune cells to migrate, attach and detach from other cells during an immune response and inflammation [38]. Dysregulation of ICAM genes can lead to chronic inflammation and immune dysfunction, which are common features of aging and many chronic diseases. The results of our study have revealed that expression levels of the ICAM4 gene are higher in the older age group and agree with other studies showing that the expression of ICAM genes is increased in different age-related diseases such as atherosclerosis [39], cellular senescence [39–41] and even Alzheimer’s disease [42].

GPAM is involved in the metformin pathway [43], a drug often prescribed to treat type 2 diabetes, and a well-studied senomorphic drug. Studies suggest that beyond its lifespan-promoting activity, metformin may be able to delay the onset of age-related diseases, such as neurodegenerative and cardiovascular, likely by acting on key aging hallmarks, such as inflammation, autophagy, and cellular senescence [44]. In addition, metformin may be able to extend lifespan and reduce the risk of age-related diseases due to its anti-hyperglycemic properties, by improving the sensitivity to insulin and metabolic health as well as reduction of oxidative stress and by protecting endothelial and vascular function [45].

The GATSL3 gene encodes a protein of the same name which is an arginine sensor for the mTORC1 (mechanistic target of rapamycin complex 1) pathway a well-known regulator of aging and lifespan. GATSL3 is a negative regulator of mTORC1 [46] and inhibition of mTORC1 has been shown to increase lifespan and confer protection against age-related pathologies [47]. The mechanisms by which mTOR regulates aging and lifespan are not clear but inhibition of mTOR has been shown to suppress the senescence-associated secretory phenotype [48]. In our study GATSL3 displayed a biphasic relationship with age, presenting a maximum expression in the middle-aged group [60–70] and minimal expression in the older age group (> 70). Taken together, GATSL3 as a negative regulator of mTORC1 signaling might have implications for potential therapeutic interventions for age-related diseases.

### Multiomic data integration for age-related cognitive alterations

Pathway enrichment analysis based on the multi-omics signature was biased towards variables from the SNP dataset, owing to their preselection from the Polygenic risk model. Nevertheless, our integrated omics approach reinforces the involvement of synaptic dysfunction, axonal degeneration, microtubule cytoskeleton organization, and glycosylation in cognitive aging. Contrary to neurodegenerative disorders, minimal neuronal loss occurs during normal aging, instead age-related cognitive alteration seems to be related to changes in neural plasticity and a result of region-specific morphological changes in dendritic branching and spine density, which ultimately lead to a progressive reduction in synaptic density and synaptic transmission [49]. Moreover, age-related structural changes in white matter (WM), including reductions in WM volume, accumulation of WM lesions, and disrupted WM integrity, have all been associated with aging and age-related cognitive deficits [6]. White matter is mainly composed of myelinated long-distance axonal projections and a decline in WM integrity may reflect demyelination and axonal degeneration. Cytoskeletal remodeling plays a significant role in maintaining axonal integrity, axonal transport, driving myelination, and regulating dendritic spine morphology and synaptic plasticity [50, 51]. Furthermore, loss of cytoskeletal integrity has been associated with aging and various diseases, including Alzheimer's disease. Consequently, there is ongoing development of agents targeting cytoskeletal integrity for potential therapeutic interventions [49].

Our findings also provide evidence supporting the involvement of glycosylation in cognitive aging. Glycosylation, the most common post-translational modification of proteins and lipids, is an essential process for normal biological functioning. It plays a vital role in the development and function of the nervous system, including plasticity and memory formation [52–54]. Consequently, it is not surprising that nearly all congenital disorders of glycosylation (CDGs) exhibit neurological symptoms such as brain atrophy, cognitive impairment, and delayed intellectual development 55. Also, disrupted glycosylation has been implicated in the pathogenesis of neurodegenerative disorders such as Alzheimer's and Parkinson’s diseases [56, 57].

Enhancing our comprehension of the biological processes accountable for the deterioration of cognition due to aging is undeniably an urgent matter of public health. Our findings expand on previous research by indicating that phenotypic correlations between neurocognitive and white-matter integrity might be partly driven by shared genetic factors and emphasizing the molecular mechanism behind these correlations. The identification of non-linear patterns in age-related alterations serves as a compelling testament to the intricate molecular complexity underlying the aging process. These findings not only challenge the notion of a straightforward linear progression but also illuminate a critical period of physiological transformation during the aging process.

The integration of multi-omic sdata has yielded significant findings, revealing the existence of numerous interconnected biological pathways that contribute to age-related cognitive performance in memory. Among these pathways, structural neuroplasticity has emerged as a prominent factor responsible for this decline. Notably, the identification of concordant enrichment terms between genomic, peripheral transcriptomics and methylation data not only supports the reliability of using peripheral cells, in particular PBMCs, as proxies for brain-related studies but also reinforces the notion that systemic changes in peripheral tissues might reflect the molecular changes occurring in the brain during aging. These findings offer a valuable opportunity to leverage accessible and less invasive samples for monitoring and potentially developing interventions for age-related cognitive performance.

However, our study has several limitations to consider. First of all, the cohort at hand was randomly selected from specific local health authority registries in the north of Portugal and consisted of individuals of European ancestry. This demographic restriction may hinder generalizability of our finding to other populations with different genetic backgrounds and demographics. Secondly, due to sample availability and quality, the subset of participants included in the methylation and transcriptomics analyses does not accurately represent the larger cohort in terms of the distribution of females and males (transcriptomic data) and proportion of individuals in each age group (both for transcriptomic and methylation data). Consequentially, the interpretation of these results should be approached with caution, as they may not be generalizable and may have reduced statistical power. Thirdly, our study used a cross-sectional design, which captures data at a single point in time, to assess age-related cognitive, methylation and transcriptomic alteration. This design limits the ability to establish causal relationships and draw longitudinal conclusions. It is important to acknowledge that further research utilizing longitudinal designs is needed to provide more robust evidence. Fourthly the study used a relatively small cohort, particularly when examining age-related methylation and transcriptomic alterations, as well as integrating multi-omics data. A larger sample size would enhance the reliability and generalizability of the results. Additionally, in high-dimensional omics datasets where the number of variables exceeds the number of samples, the risk of overfitting exists. Therefore, it is crucial to validate these results using an external cohort to ensure their accuracy and reliability. In summary, while our study provides valuable insights, it is essential to recognize and consider these limitations when interpreting the findings. Future research addressing these limitations will contribute to a more comprehensive understanding of age-related cognitive alterations.

## Conclusions

In conclusion, our study offers a comprehensive exploration of the intricate mechanisms driving age-related cognitive performance. Notably, our findings highlight shared genetic risk factors and potential common pathophysiological pathways, underscoring the phenotypic association between white matter integrity and age-related cognitive performance. These associations likely involve multifaceted mechanisms influenced by genetic susceptibility to various vascular and non-vascular effects. Moreover, our observation of non-linear patterns in age-related alterations in methylation and transcriptomic profiles challenges the simplistic view of aging progression, emphasizing the complex molecular nature of aging. Additionally, the integration of multi-omics data reveals interconnected biological pathways contributing to age-related cognitive performance, with structural neuroplasticity emerging as a significant factor. The consistency of enrichment terms across genomic, peripheral transcriptomics, and methylation data underscores the potential utility of peripheral cells as proxies for brain-related studies, offering avenues for less invasive monitoring and potential interventions for age-related cognitive performance. However, our study has limitations, including the demographic homogeneity of our cohort and the cross-sectional design. Additionally, the relatively small sample size and risk of overfitting in omics datasets warrant validation in larger cohorts. Despite these limitations, our findings contribute valuable insights into the biological underpinnings of age-related cognitive performance. Future research addressing these limitations and employing longitudinal designs will enhance our understanding and inform interventions to promote healthy aging and preserve cognitive function.

## Methods

### Cohort characterization

The study cohort (*n* = 443, after exclusion criteria) hereon after designated as Minho cohort is part of a larger cohort randomly selected from the Guimarães and Vizela local health authority registries in the north of Portugal [58]. Table 1 provides descriptive statistics of the Minho aging cohort. Selection criteria are described elsewhere [59]. Primary exclusion criteria included participants diagnosed with neuropsychiatric disorder and/or dementia, who had a stroke, renal failure, or overt thyroid pathology, were unable to understand informed consent or chose to withdraw from the study. A team of experienced clinicians performed a standardized clinical interview.

### Neurocognitive evaluation

Participants underwent a thorough neurocognitive evaluation conducted by a team of trained psychologists. Tests included measures of global cognition (Mini-Mental State Examination (MMSE) [60]; short-term verbal memory and attention were assessed using the Digit Span Test (parameters; digits span forward and backward) [61]; verbal learning and memory storage, retention and recall (The Buschke Selective Reminding Test parameters (SRT): consistent long term retrieval (CLTR), long term storage (LTS), delayed recall and intrusions [62]; inhibition/cognitive flexibility were assessed using the Stroop Color and Word test (parameters: words, colors and words/colors) [63]; verbal/phonetic fluency assessed by the Controlled Oral Words association test F-A-S (COWAT-FAS parameters: admissible and non-admissible) [64].

### Imaging data acquisition

The details of imaging data acquisition are comprehensively described in [65]. In summary, MRI scans were conducted using a Siemens Magnetom Avanto 1.5 T (Siemens Medical Solutions, Erlangen, Germany) scanner with a 12-channel receive-only head coil. Imaging session included a single structural T1 sequence, specifically a 3D MPRAGE (magnetization prepared rapid gradient echo) sequence, with the following parameters: a repetition time (TR) of 2.730 s, an echo time (TE) of 3.48 ms, 176 sagittal slices with no gaps, a flip angle (FA) of 7°, an in-plane resolution of 1.0 mm × 1.0 mm, and a slice thickness of 1.0 mm. The Freesurfer toolkit version 5.1 (https://surfer.nmr.mgh.harvard.edu), operating on an Ubuntu 12.04 LTS system, was utilized for the segmentation of brain cortical and subcortical structures during structural analysis. The software employs a semi-automated segmentation workflow, which includes stages such as spatial registration to the Talairach standard space, skull removal, normalization of white matter intensity, and tessellation of gray matter-white matter segmentation. Two atlases were employed for cortical parcellation: one gyral-based atlas defining 68 structures [66], and another that includes both gyral and sulcal regions, defining 148 distinct brain areas [67]. The study considered subcortical, white matter, and gyral-based cortical segmentations of 106 participants. For descriptive statistics on the imaging dataset, please refer to supplementary Table 5.

### OMICS sample processing

Genomic DNA/RNA were extracted from PBMCs (for methylation and transcriptomic analysis) or whole blood (for genotyping) using a commercially available kit according to the manufacturer's protocol (All prep DNA/RNA kit, Qiagen). DNA integrity assessment was done using gel electrophoresis and quantified using Qubit dsDNA HS (High Sensitivity) Assay Kit measured on the Qubit Fluorometer (Life Technologies, Carlsbad, CA). RNA quality was assessed using Bioanalyzer RNA 6000 Nano kit (Agilent), 6 out of 107 samples were excluded due to bad RNA quality.

### Genetic data

Genotypic data were collected for 564 individuals using the Illumina Neuro Consortium Array (Infinium Core-24 + v1.2). Bioinformatics quality control (QC) was performed using Plink v1.9 software according to standard protocols [68]. Briefly, after exporting samples with call rate per marker ≥ 95% from Illumina-designed software GenomeStudio, per-sample QC involved controlling for discrepancies between reported and genotypic sex, heterozygosity rate, identification of related individual and identification of individuals of divergent ancestry. Per-SNP QC consisted of excluding SNP with excessive missing genotypes (call rate > 0.05), the deviation for Hardy‐Weinberg Equilibrium (HWE > 0.00001), and Minor Allele Frequency (MAF < 0.01). After QC, genotypes were imputed using the Michigan imputation server [69] with the Haplotype Reference Consortium (HRC) reference panel (version r1.1 2016). The imputed genotypes were then subjected to the same QC filters as previously described. Tree sample were excluded duo to a misidentified ID. A total of 418 samples (after exclusion criteria) and 5,389,594 SNPs passed QC and were used in subsequent analysis.

### Transcriptomic data

A subset of randomly selected 101 RNA samples was used for gene expression profiling. Samples were processed with the SurePrint G3 Human Gene Expression v3 8 × 60 K arrays and scanned in an Agilent scanner. The microarray contained more than 60,000 probe sets representing approximately 37,700 known transcripts, allowing the analysis of over 26,000 genes. Labeling was performed using the Low Input Quick Amp Labeling Kit, One-Color (Agilent Technologies). The analysis was performed using R (v4.0.2) and the computing environment package, limma (v3.44.3) [70]. The quality of the data was assessed using multi-dimensional scaling analysis (MDS) [70], 6 outliers were detected and removed from further analysis. In addition, one sample was excluded duo to a misidentified ID. The characteristics of subsamples from the remaining 94 samples are documented in Supplementary Table 5. Among these 94 samples, 79 samples included genomic data, 40 had methylation data, and 38 possessed both genomic, and methylation data.

### Methylation data

A subset of randomly selected 41 DNA samples were processed with the Infinium Methylation EPIC Bead Chip (Illumina, Inc), according to the manufacturer’s instructions. Among these 41 samples, 38 included genomic and transcriptomic data, 2 had exclusively transcriptomic data and 1 had exclusively genomic data.

Subsamples characteristics are present in Supplementary Table 5. The quality control, preprocessing, and normalization of the DNA methylation data were analyzed using R (v4.1.1) packages minfi (v1.38.0). FlowSorted. Blood. EPIC (v2.0.0). Peripheral blood cell composition (relative proportion) was estimated [71] with raw data. Methylation was normalized with the quantile normalization method and deconvolution of the blood cells was performed to reduce the statistical bias in subsequent analysis. Probes with poor quality, rowSums(detP > 0.01), located between SNPs, cross-reactive, related to the differences in gender (autosomal and sex chromosomes) were identified and removed.

### Genome-wide polygenic risk scores (PRS)

Genome-wide PRS were constructed for each healthy aging Minho cohort participant using the “standard weighted allele” method implemented in PRSice-2 software [72]. SNPs were weighted by their GWAS effect sizes on MRI markers of cerebral small vessel disease (white matter hyperintensities; WMH, fractional anisotropy; FA and mean diffusivity; MD). GWAS summary statistics for WMH (*N* = 18,381), FA (*N* = 17,663), and MD (*N* = 17,467) [73], were obtained via the Cerebrovascular Disease Knowledge Portal (https://cd.hugeamp.org/) data download page (http://www.kp4cd.org/dataset_downloads/stroke). Genome-wide PRS was used to explore the association between WMH, FA, and MD with age-related cognitive outcomes using the additive model while adjusting for age, sex, school years, and 6 ancestral PCA via linear regression. Linkage disequilibrium (LD) clumping was performed under default settings (250 kb window, r2 < 0.1). To accurately predict the outcome of interest, and avoid overfitting and Type-I error, the best threshold was identified by computing different genome-wide PRSs and calculating empirical p-values (pE) using 10,000 permutations [72] Furthermore, each permutation test provided a Nagelkerke’s pseudo r2 after adjustment for study-specific PCs 1–6, age, sex and school years as covariates. The “best-fit PRS” was identified as the most associated with the target trait across the range of P-value thresholds considered. As an added measure, an FDR correction was applied to account for the three base MRI traits and the two cognitive outcomes tested. The list of variants used for PRS calculation was obtained using the –print-snp flag from PRSice software. Subsequently, g: Profiler [https://biit.cs.ut.ee/gprofiler/] web server was used to map SNP rs-codes, that overlap with at least one protein-coding Ensembl gene, to obtain gene names.

### Sparse partial least squares-discriminant analysis (sPLS-DA)

Sparse partial least squares-discriminant analysis (sPLS-DA) was performed separately on cognitive, imaging, methylation, and transcriptomic data to whether identify individual signatures best discriminating [50–60],  [60–70], [70-…] age groups or cognitive profiles using the mixOmics R package [19]. Each cognitive test was categorized into “low” and “high” performers based on mean calculation, utilizing a threshold determined by the mean value. sPLS-DA performs dimension reduction and variable selection in a supervised classification setting in one step. It is based on Partial Least Squares regression (PLS), a multivariate linear regression method that can deal with high-dimensional data and collinearity among predictors (eg. the inherent correlation among neurocognitive tests) but using a sparse penalty for variable selection (which penalizes the number of predictors favoring the ones that explain most of the variance of the data). The optimal number of components onto which the data is projected, and the optimal number of features to select on each component are determined by tuning these parameters using a cross-validation procedure and by assessing the overall error rate. In this study, the number of components and features were identified using leave-one-out cross-validation.

### Data integration

**Fig. 5:**
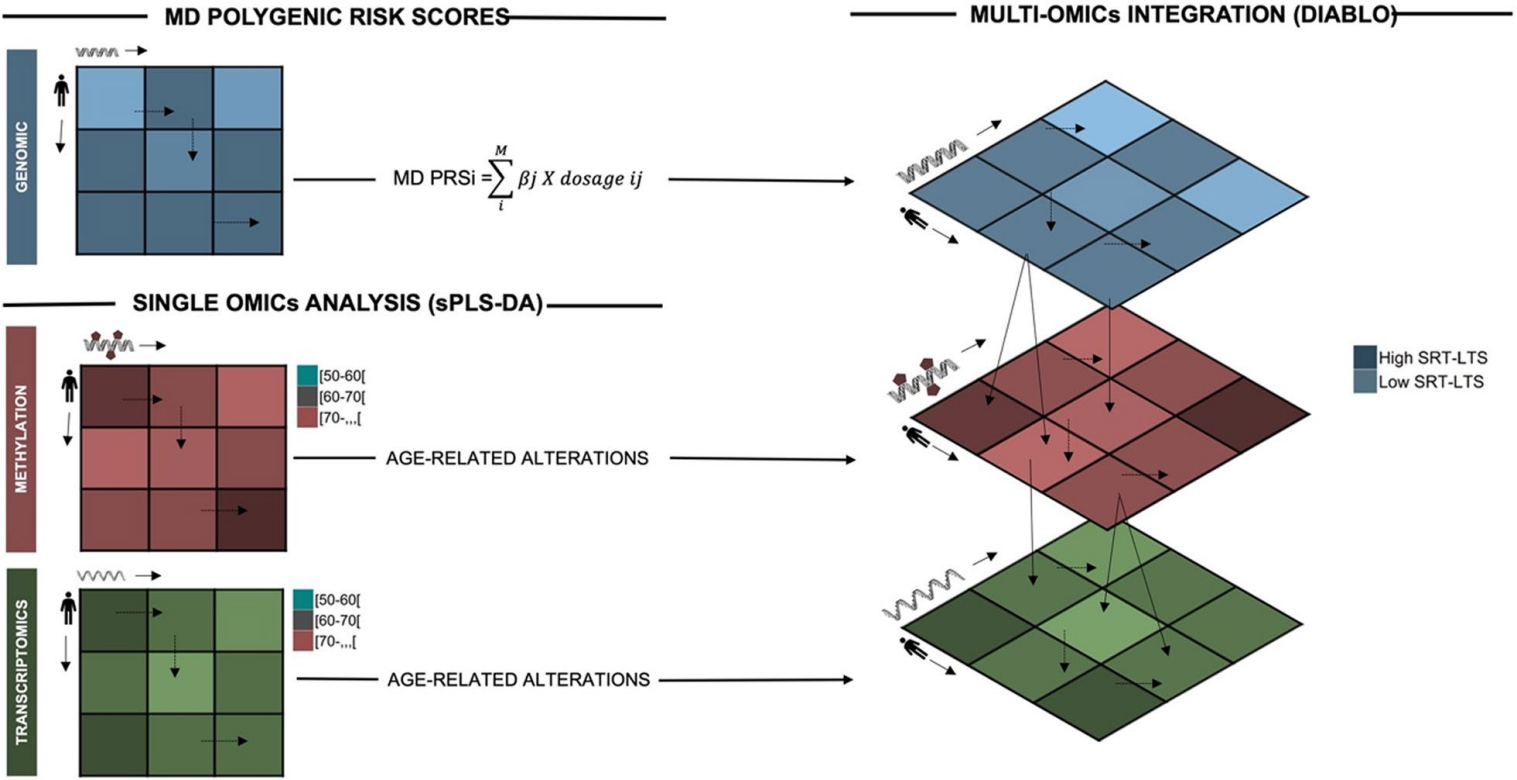
Multi-omic data integration pipeline Single nucleotide polymorphisms used in the construction of the MD-PRS model (1383) together with age-related methylation (350 CpG) and transcriptomic (58 genes) variables, pre-selected using Sparse partial least squares-discriminant analysis (sPLS-DA), were integrated using the DIABLO model (MixOmics R package). Multi-omic data integration was achieved by identifying a set of correlated variables, both within and between the different omic layers, that best discriminate individuals with higher and lower performance in the SRT-LTS

Integration of age-related methylation sites (350 CpG) transcriptomic (58 genes) variables and SNPs used in the construction of the polygenic model (1383 SNP) of 38 matching samples, 16 with low and 22 with high SRT-LTS, respectively (for descriptive statistics on the multi-omics dataset, please refer to supplementary Table 5), was conducted to identify correlated omics features discriminating individuals with high and low SRT-LTS scores (Fig. 5). The analysis was performed using the Data Integration Analysis for Biomarker Discovery using the Latent cOmponents (DIABLO) [74] algorithm implemented in the mixOmics R package [19]. The model, also known as multiblock sPLS-DA, is an extension of the previously described sPLS-DA and it incorporates rCCA (Regularized Canonical Correlation Analysis) as the integration method. During the integration process, Diablo performs variable selection in a supervised fashion to identify the shared variation across the different omics datasets while accounting for the potential correlations and relationships between variables within and between the omics layers. Therefore, like sPLS-DA, the method requires parameter tuning. Parameter tuning, for number of components and number of variables, was performed as described above in the sPLS-DA approach. In addition, an input design matrix to define the correlation between each omics data set is needed. A design matrix of 0.1 was chosen to prioritize the discrimination between groups (High vs Low SRT-LTS scores).

### Pathway enrichment analysis

Pathway enrichment analysis was performed using the web-based Metascape portal [http://metascape.org] [20] with the following ontology sources: GO Biological Processes, GO Molecular Functions, GO Cellular Components, KEGG pathways, Reactome and Transcriptional Factor Targets gene sets. Terms with *p*-values < 0.01, a minimum of 3 counts, and an enrichment factor > 1.5 (ratio between the observed counts and the counts expected by chance) were hierarchically clustered based on similarities (Kappa scores) among their gene memberships. Enriched terms with a Kappa score > 0.3 were considered a cluster and the most significant term within each cluster was selected to represent the entire cluster. An adjusted *p*-value (q-values < 0.05), using Benjamini–Hochberg false-discovery rate to account for multiple testing, was considered statistically significant.

### Supplementary Information

**Additional file 1.**

**Additional file 2.**

**Additional file 3.**

**Additional file 4.**

**Additional file 5.**

**Additional file 6.**

**Additional file 7.**

**Additional file 8.**

**Additional file 9.**

**Additional file 10.**

## Data Availability

The datasets generated during the current study are available from the corresponding author on reasonable request and upon Data Usage Agreement.

## References

[1] Grady C (2012). The cognitive neuroscience of ageing (trends in neurocognitive aging). Nat Rev Neurosci.

[2] Kaup AR, Mirzakhanian H, Jeste DV, Eyler LT (2011). A review of the brain structure correlates of successful cognitive aging. J Neuropsychiatry Clinic Neurosci..

[3] Oschwald J (2019). Brain structure and cognitive ability in healthy aging: a review on longitudinal correlated change. Rev Neurosci.

[4] Klostermann EC, Braskie MN, Landau SM, O’Neil JP, Jagust WJ (2012). Dopamine and frontostriatal networks in cognitive aging. Neurobiol Aging.

[5] Finkel D, Reynolds CA, McArdle JJ, Pedersen NL (2007). Age changes in processing speed as a leading indicator of cognitive aging. Psychol Aging.

[6] Bennett IJ, Madden DJ (2014). Disconnected aging: cerebral white matter integrity and age-related differences in cognition. Neuroscience.

[7] Wardlaw JM, Smith C, Dichgans M (2019). Small vessel disease: mechanisms and clinical implications. Lancet Neurol.

[8] Wardlaw JM, Valdés Hernández MC, Muñoz-Maniega S (2015). What are white matter hyperintensities made of? Relevance to vascular cognitive impairment. J Am Heart Assoc.

[9] Melzer D, Pilling LC, Ferrucci L (2019). The genetics of human ageing. Nat Rev Genet.

[10] Watanabe K (2019). A global overview of pleiotropy and genetic architecture in complex traits. Nat Genet.

[11] Choi SW, Shin T, Mak H, Reilly PFO (2021). A guide to performing polygenic risk score analyses Introduction to polygenic risk scores. Nat Protoc.

[12] International Schizophrenia Consortium; Purcell SM, Wray NR, Stone JL, Visscher PM, O'Donovan MC, Sullivan PF, Sklar P. Common polygenic variation contributes to risk of schizophrenia and bipolar disorder. Nature. 2009;460(7256):748-52. 10.1038/nature08185. 10.1038/nature08185 PMC3912837 19571811

[13] McIntosh AM (2013). Polygenic risk for schizophrenia is associated with cognitive change between childhood and old age. Biol Psychiatry.

[14] Hagenaars SP (2016). Polygenic risk for coronary artery disease is associated with cognitive ability in older adults. Int J Epidemiol.

[15] Edwards SL, Beesley J, French JD, Dunning M (2013). Beyond GWASs: illuminating the dark road from association to function. Am J Hum Genet.

[16] Xiao FH, Wang HT, Kong QP (2019). Dynamic DNA methylation during aging: a “prophet” of age-related outcomes. Front Genet.

[17] Song X (2021). Transcriptomics analysis reveals shared pathways in peripheral blood mononuclear cells and brain tissues of patients with schizophrenia. Front Psychiatry.

[18] López-Otín C, Blasco MA, Partridge L, Serrano M, Kroemer G (2023). Hallmarks of aging: an expanding universe. Cell.

[19] Rohart F, Gautier B, Singh A, Lê Cao KA (2017). mixOmics: an R package for ‘omics feature selection and multiple data integration. PLoS Comput Biol.

[20] Zhou Y (2019). Metascape provides a biologist-oriented resource for the analysis of systems-level datasets. Nat Commun.

[21] Salthouse TA (1996). The processing-speed theory of adult age differences in cognition. Psychol Rev.

[22] Coupé P, Manjón JV, Lanuza E, Catheline G (2019). Lifespan changes of the human brain In Alzheimer’s disease. Sci Rep.

[23] Reuben A, Brickman AM, Muraskin J, Steffener J, Stern Y (2011). Hippocampal atrophy relates to fluid intelligence decline in the elderly. J Int Neuropsychol Soc.

[24] O’Shea A, Cohen RA, Porges EC, Nissim NR, Woods AJ (2016). Cognitive aging and the hippocampus in older adults. Front Aging Neurosci.

[25] Papp KV (2014). Processing speed in normal aging: Effects of white matter hyperintensities and hippocampal volume loss. Aging Neuropsychol Cognit.

[26] Charlton RA (2006). White matter damage on diffusion tensor imaging correlates with age-related cognitive decline. Neurology.

[27] De Groot M (2013). Changes in normal-appearing white matter precede development of white matter lesions. Stroke.

[28] Merenstein JL, Corrada MM, Kawas CH, Bennett IJ (2021). Age affects white matter microstructure and episodic memory across the older adult lifespan. Neurobiol Aging.

[29] Carmeliet P, Tessier-Lavigne M (2005). Common mechanisms of nerve and blood vessel wiring. Nature.

[30] Cao L (2004). VEGF links hippocampal activity with neurogenesis, learning and memory. Nat Genet.

[31] Fjell AM (2013). Critical ages in the life course of the adult brain: nonlinear subcortical aging. Neurobiol Aging.

[32] Usselman CWNSSJRB (2017). A meta-analysis of heritability of cognitive aging: minding the “missing heritability” gap. Physiol Behav.

[33] Cao K, Ryvkin P, Hwang YC, Johnson FB, Wang LS (2013). Analysis of nonlinear gene expression progression reveals extensive pathway and age-specific transitions in aging human brains. PLoS ONE.

[34] Sturm G (2019). Human aging DNA methylation signatures are conserved but accelerated in cultured fibroblasts. Epigenetics.

[35] Vershinina O, Bacalini MG, Zaikin A, Franceschi C, Ivanchenko M (2021). Disentangling age-dependent DNA methylation: deterministic, stochastic, and nonlinear. Sci Rep.

[36] Lehallier B (2019). Undulating changes in human plasma proteome profiles across the lifespan. Nat Med.

[37] Bergman A, Atzmon G, Ye K, MacCarthy T, Barzilai N (2007). Buffering mechanisms in aging: a systems approach toward uncovering the genetic component of aging. PLoS Comput Biol.

[38] Guerra-Espinosa C, Jiménez-Fernández M, Sánchez-Madrid F, Serrador JM (2024). ICAMs in immunity intercellular adhesion and communication. Cells.

[39] Gorgoulis VG (2005). p53-dependent ICAM-1 overexpression in senescent human cells identified in atherosclerotic lesions. Lab Invest.

[40] Shelton DN, Chang E, Whittier PS, Choi D, Funk WD (1999). Microarray analysis of replicative senescence. Curr Biol.

[41] Minamino T (2002). Endothelial cell senescence in human atherosclerosis: role of telomere in endothelial dysfunction. Circulation.

[42] Akiyama H (1993). Expression of intercellular adhesion molecule (ICAM)-1 by a subset of astrocytes in Alzheimer disease and some other degenerative neurological disorders. Acta Neuropathol.

[43] Gong L, Goswami S, Giacomini KM, Altman RB, Klein TE (2012). Metformin pathways: pharmacokinetics and pharmacodynamics. Pharmacogenet Genom.

[44] Cheng FF, Liu YL, Du J, Lin JT (2022). Metformin’s mechanisms in attenuating hallmarks of aging and age-related disease. Aging Dis.

[45] Mohammed I, Hollenberg MD, Ding H, Triggle CR (2021). A critical review of the evidence that metformin is a putative anti-aging drug that enhances healthspan and extends lifespan. Front Endocrinol.

[46] Chantranupong L (2016). The CASTOR proteins are arginine sensors for the mTORC1 pathway. Cell.

[47] Johnson SC, Rabinovitch PS, Kaeberlein M (2013). MTOR is a key modulator of ageing and age-related disease. Nature.

[48] Herranz N (2015). mTOR regulates MAPKAPK2 translation to control the senescence-associated secretory phenotype. Nat Cell Biol.

[49] Burke SN, Barnes CA (2006). Neural plasticity in the ageing brain. Nat Rev Neurosci.

[50] Prokop A (2013). The intricate relationship between microtubules and their associated motor proteins during axon growth and maintenance. Neural Dev.

[51] Jaworski J (2009). Dynamic microtubules regulate dendritic spine morphology and synaptic plasticity. Neuron.

[52] Varki A (2017). Biological roles of glycans. Glycobiology.

[53] Iqbal S, Fard MG, Everest-Dass A, Packer NH, Parker LM (2018). Understanding cellular glycan surfaces in the central nervous system. Biochem Soc Trans.

[54] Scott H, Panin VM (2014). The role of protein N-glycosylation in neural transmission. Glycobiology.

[55] Conroy LR, Hawkinson TR, Young LEA, Gentry MS, Sun RC (2021). Emerging roles of N-linked glycosylation in brain physiology and disorders. Trends Endocrinol Metabol.

[56] Yuzwa SA (2012). Increasing O-GlcNAc slows neurodegeneration and stabilizes tau against aggregation. Nat Chem Biol.

[57] Marotta NP (2015). O-GlcNAc modification blocks the aggregation and toxicity of the protein α-synuclein associated with Parkinson’s disease. Nat Chem.

[58] Santos NC (2013). Mood is a key determinant of cognitive performance in community-dwelling older adults: a cross-sectional analysis. Age.

[59] Paulo AC (2011). Patterns of cognitive performance in healthy ageing in northern Portugal: a cross-sectional analysis. PLoS ONE.

[60] Guerreiro M (1994). Adaptação à população portuguesa da tradução do “mini mental state examination” (MMSE). Rev Port Neurol.

[61] Wechsler D (1997). WAIS-III: wechsler adult intelligence scale.

[62] Buschke H, Sliwinski M, Kuslansky G, Lipton RB (1995). Aging, encoding specificity, and memory change in the double memory test. J Int Neuropsychol Soc.

[63] Strauss E, Sherman EMS, Spreen O. A compendium of neuropsychological tests: Administration, norms, and commentary 3rd ed. Oxford University Press; 2006.

[64] Lezak M, Howieson D, Loring D (2004). Neuropsychological assessment.

[65] Soares JM, Marques P, Magalhães R, Santos NC, Sousa N (2014). Brain structure across the lifespan: the influence of stress and mood. Front Aging Neurosci.

[66] Desikan RS (2006). An automated labeling system for subdividing the human cerebral cortex on MRI scans into gyral based regions of interest. Neuroimage.

[67] Destrieux C, Fischl B, Dale A, Halgren E (2010). Automatic parcellation of human cortical gyri and sulci using standard anatomical nomenclature. Neuroimage.

[68] Anderson CA (2010). Data quality control in genetic case-control association studies. Nat Protoc.

[69] Das S (2016). Next-generation genotype imputation service and methods. Nat Genet.

[70] Ritchie ME (2015). Limma powers differential expression analyses for RNA-sequencing and microarray studies. Nucleic Acids Res.

[71] Houseman EA (2012). DNA methylation arrays as surrogate measures of cell mixture distribution. BMC Bioinformatics.

[72] Choi SW, O’Reilly PF (2019). PRSice-2: polygenic risk score software for biobank-scale data. Gigascience.

[73] Persyn E (2020). Genome-wide association study of MRI markers of cerebral small vessel disease in 42,310 participants. Nat Commun.

[74] Singh A (2019). DIABLO: an integrative approach for identifying key molecular drivers from multi-omics assays. Bioinformatics.

